# Development of high-lysine rice via endosperm-specific expression of a foreign *LYSINE RICH PROTEIN* gene

**DOI:** 10.1186/s12870-016-0837-x

**Published:** 2016-06-29

**Authors:** Xin Liu, Cuicui Zhang, Xiurong Wang, Qiaoquan Liu, Dingyang Yuan, Gang Pan, Samuel S. M. Sun, Jumin Tu

**Affiliations:** Institute of Crop Science, College of Agriculture and Biotechnology, Zhejiang University, Yuhangtang Road 866, Hangzhou, 310058 China; UGC-AoE Plant and Fungal Biotechnology Center, Department of Biology, The Chinese University of Hong Kong, Hong Kong, China

**Keywords:** Lysine, *LYSINE RICH PROTEIN* (*LRP*), Rice (*Oryza sativa* L.), High-lysine rice

## Abstract

**Background:**

Lysine (Lys) is considered to be the first limiting essential amino acid in rice. Although there have been extensive efforts to improve the Lys content of rice through traditional breeding and genetic engineering, no satisfactory products have been achieved to date.

**Results:**

We expressed a *LYSINE-RICH PROTEIN* gene (*LRP*) from *Psophocarpus tetragonolobus* (L.) DC using an endosperm-specific *GLUTELIN1* promoter (GT1) in Peiai64S (PA64S), an elite photoperiod-thermo sensitive male sterility (PTSMS) line. The expression of the foreign LRP protein was confirmed by Western blot analysis. The Lys level in the transgenic rice seeds increased more than 30 %, the total amount of other amino acids also increased compared to wild-type. Persistent investigation of amino acids in 3 generations showed that the Lys content was significantly increased in seeds of transgenic rice. Furthermore, Lys content in the hybrid of the transgenic plants also had an approximate 20 % increase compared to hybrid control. At the grain-filling stage, we monitored the transcript abundance of many genes encoding key enzymes involved in amino acid metabolism, and the results suggested that reduced amino acid catabolism led to the accumulation of amino acids in the transgenic plants. The genetically engineered rice showed unfavorable grain phenotypes compared to wild-type, however, its hybrid displayed little negative effects on grain.

**Conclusions:**

Endosperm-specific expression of foreign *LRP* significantly increased the Lys content in the seeds of transgenic plant, and the the Lys increase was stably heritable with 3 generation investigation. The hybrid of the transgenic plants also showed significant increases of Lys content in the seeds. These results indicated that expression of *LRP* in rice seeds may have promising applications in improving Lys levels in rice.

**Electronic supplementary material:**

The online version of this article (doi:10.1186/s12870-016-0837-x) contains supplementary material, which is available to authorized users.

## Background

Rice is a staple food for more than half of the world’s population and the main protein source for billions of people worldwide, especially in less developed areas. Similarly, rice can also be the main component of livestock feed and a major source of protein for animals. However, the protein in rice is nutritionally incomplete due to a deficiency in essential amino acids for humans and livestock [5]. Indeed, based on the report of World Health Organization in 2007, the content of lysine (Lys) in seeds is particularly low [26]. Therefore, Lys is considered to be the first limiting essential amino acid in rice. Previous studies have focused on genetic approaches for enhancing Lys levels in cereal seeds. A promising step in the improvement of Lys properties was the discovery of the *opaque2* mutant, which shows significant increases in kernel Lys and tryptophan (Trp) [19, 20]. A subsequent improved maize variety of the *opaque*2 genotype, quality protein maize (QPM), has been developed [8], which not only significantly increased the Lys level but also solved the problem of the negative effects on important agronomical traits that accompany the classical *opaque2* mutation. Thus, QPM is regarded as promising commercial material for improving the Lys balance. Unfortunately, many attempts to breed similar genotypes in other cereals have not achieved the desired results. Therefore, additional approaches to improve Lys levels are urgently needed.

Lys biosynthesis in plants occurs via a pathway of aspartate (Asp) catabolism followed by the conversion of aspartate semialdehyde to dihydrodipicolinate, which is catalyzed by dihydrodipicolinate synthase (DHPS), and finally to Lys through a series of steps carried out by diaminopimelate decarboxylase (DAPD) [2]. Lys is catabolized to saccharopine by Lys ketoglutaric acid reductase (LKR) and saccharopine dehydropine dehydrogenase (SDH) [6, 7, 18]. Recent advances in genetic engineering have supplied new opportunities to achieve a balanced Lys content in cereal grains. An approach for enhancing the free Lys content is to over-express key enzymes in the Lys synthesis pathway or to down-regulate the expression of enzymes in the catabolic pathway. For example, Zhu and Galili [33, 34] expressed a bacterial feedback-insensitive DHPS enzyme in Lys synthesis in an *Arabidopsis* knockout mutant lacking a bifunctional LKR and SDH enzyme for catabolism. The resulting plants exhibited greatly increased Lys content in their seeds; however, the engineered plants also showed adverse consequences to morphological traits, such as reduced seedling growth and a low seed germination rate. In addition, engineered rice plants over-expressing *AK*/*DHPS* and/or with RNA-interfered *LKR/SDH* displayed sharply increased Lys levels in leaves and seeds, with no observable changes in plant growth and seed germination [18], demonstrating that free Lys can accumulate to high levels in rice leaves and seeds by regulating Lys biosynthesis or catabolism.

Another genetic engineering approach is to express genes that encode quality proteins with balanced Lys composition in cereal grains. Indeed, the expression of a potato pollen-specific *SB401* gene in maize led to an increase in the seed Lys content, and no visible morphological changes were observed in the transgenic plants [30]. The expression of a lysine-rich binding protein (BiP), which contains 9.4 % Lys, significantly increased the Lys content in transgenic rice seeds; however, undesirable seed traits such as opaqueness and a floury phenotype were reported [24]. Furthermore, over-expressing two endogenous rice lysine-rich histone proteins resulted in an approximately 35 % increase in Lys in transgenic seeds, with other essential amino acids remaining balanced and no obvious physiological abnormalities in the transgenic rice [27]. These results indicate that it is possible to efficiently increase the accumulation of Lys in seeds by expressing proteins that contain a high amount of Lys residues.

From *Psophocarpus tetragonolobus* (L.) DC, Sun et al. [23] cloned a *LYSINE-RICH PROTEIN* (*LRP*) gene encoding the LRP protein with 10.8 % of Lys, which was 18kD of molecular mass. In the present study, we expressed the *LRP* gene under the control of an endosperm-specific promoter in PA64S, which is an elite photoperiod-thermo-sensitive male sterility (PTSMS) line grown in China. Upon transformation, one of the homozygous *LRP* lines, PA110, showed significant increases in all detected amino acids, in addition to Lys, in the endosperm. Herein, we also discuss the advantages and feasibility of high-Lys transgenic breeding in hybrid rice, avoiding through heterosis the adverse consequences of Lys accumulation on seed germination and 1000-grain weight.

## Results

### Generation of the PA64S-derived line PA110 expressing the *LRP* gene

**Fig. 1:**
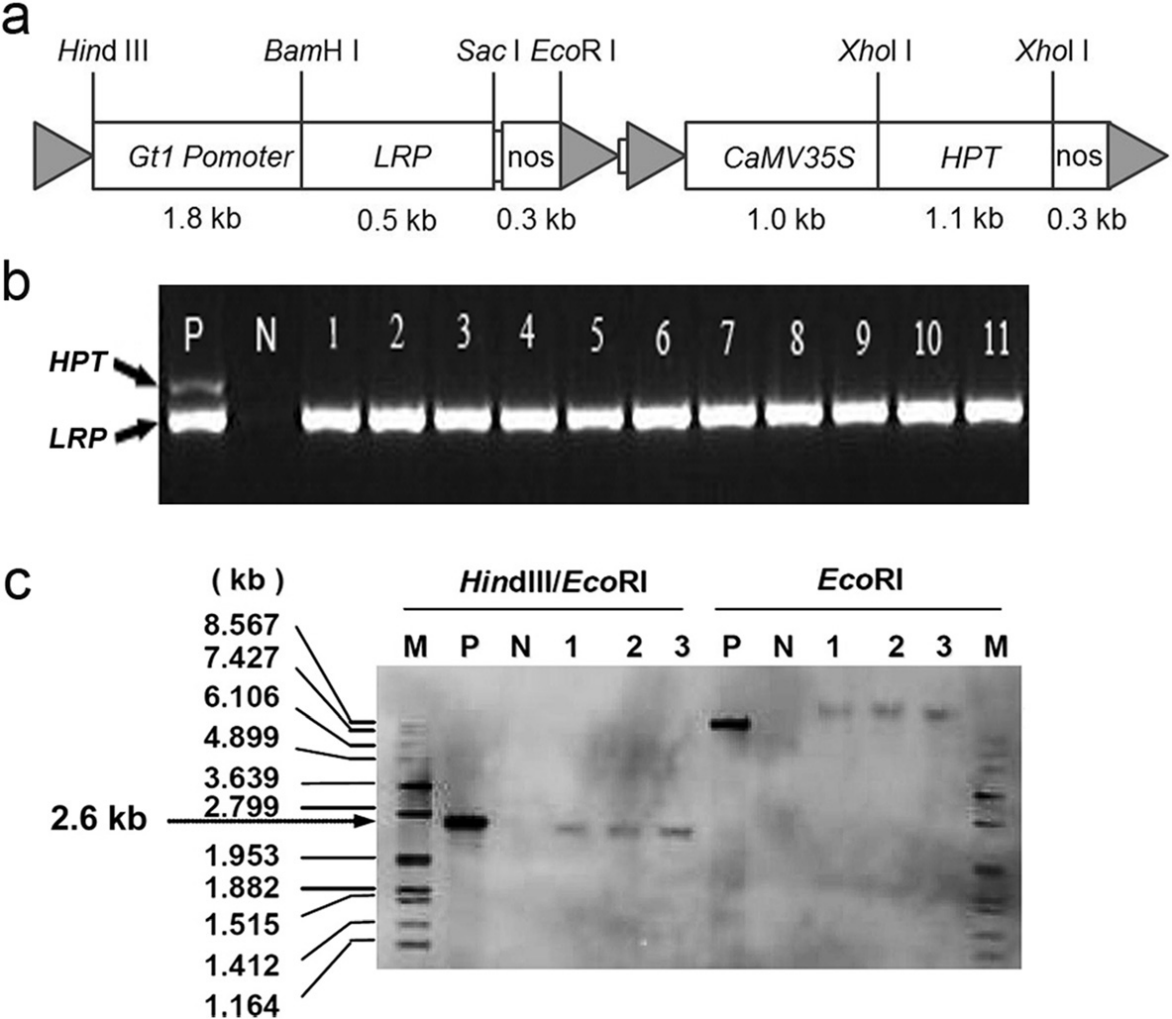
Construction of pSB130-LRP and molecular identification of transgenic plants. **a** Structure of pSB130-LRP. Empty boxes denote the GT1 promoter, *LRP*-coding region, nos terminators, *CaMV 35S* and *HPT* gene. Grey triangles denote the 4 T-DNA borders of the 2 T-DNA regions. The top panel indicates the restriction enzyme sites in the construct, and the bottom panel indicates the length of each element. **b** PCR analysis of the *HYH*-free line in the T_2_ generation. P, positive control with the pSB130-LRP plasmid; N, negative control with PA64S. Lanes 1–11 indicates 11 plants of one *LRP* transgenic line. **c** *Southern* blot analysis of the *LRP* transgenic line digested with two restriction enzymes *Hin*dIII*/Eco*RI or only *Eco*RI. Lanes 1–3 indicates 3 plants from one *LRP* transgenic line. M, P, N, denote the DNA marker, positive plasmid, and negative control with PA64S, respectively. The black arrow indicates the entire *LRP* expression cassette

To improve the Lys content of rice, a binary vector carrying the *LRP* gene under the control of the rice endosperm-specific *GLUTELIN1* promoter (GT1) and the selectable marker gene *HYGROMYCIN PHOSPHOTRANSFERASE (HPT)* driven by the cauliflower mosaic virus (CaMV) 35S promoter was introduced into PA64S via *Agrobacterium*-mediated transformation (Fig. 1a). Two independent transformants were obtained, and one homozygous line, PA64S-1-10, without the *HPT* selection marker gene was identified by multi-PCR analysis (Fig. 1b). *Eco*RI digestion of the genomic DNA following with *Southern* blot analysis revealed that this homozygous line harbors a single copy of the *LRP* gene (Fig. 1c). Furthermore, *Southern* blot analysis with *Hin*dIII and *Eco*RI double digestion released a 2.6-kb fragment corresponding to the complete *LRP* expression cassette, thus suggesting the complete integration of the *LRP* gene into the recipient genome of PA64S-1-10 (Fig. 1c). The line with the single copy of the intact *LRP* gene was named PA110 and used for further study.

### Efficient expression of *LRP* in the endosperm of PA110

**Fig. 2:**
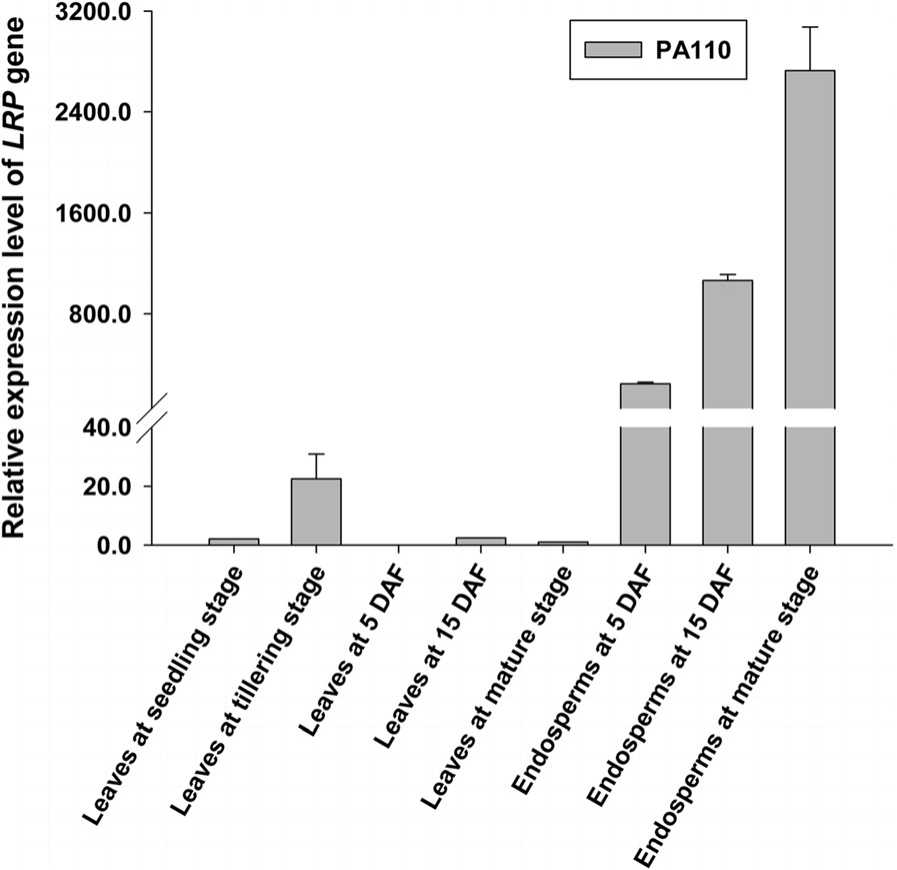
qRT-PCR analysis of the *LRP* gene in different tissues at different growth stages in PA110. Five leaf samples were prepared at the seedling, tillering, 5 DAF, 15 DAF and mature stages, and 3 endosperm samples were prepared during endosperm developing stages. Error bars indicate the s.e.m. calculated from 3 technical replicates

**Fig. 3:**
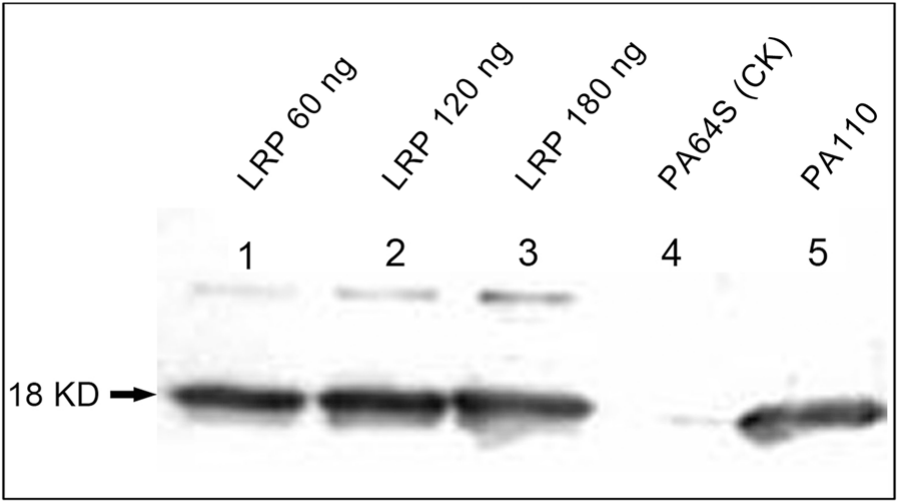
Western blot analysis of the LRP protein in PA110 seeds. Lanes 1–3 indicate 60 ng, 120 ng, and 180 ng of purified LRP protein extracted from the seeds of *Psophocarpus tetragonolobus* (L.) DC. Lanes 4 and 5 are proteins extracted from mature PA64S and PA110 seeds, respectively. An arrow indicates the LRP protein at the molecular mass of 18 kDa

To verify the expression pattern of the GT1 promoter, we monitored *LRP* transcripts by quantitative real time-PCR (qRT-PCR). The results showed dramatic increases in *LRP* transcripts in endosperms during grain filling, but only a small amount was detected in leaves from the seedling to mature stages (Fig. 2). This result demonstrated that GT1-driven *LRP* was indeed expressed in an endosperm-specific manner. Western blot analysis was employed to evaluate the expression of the LRP protein in the endosperm of PA110; known concentrations of purified LRP from *Psophocarpus tetragonolobus* (L.) DC seeds were used as quantification references, and soluble protein extracted from wild-type PA64S was used as a negative control. The results revealed a band with the expected molecular mass of approximately 18 kDa in the PA110 sample (Fig. 3), which confirmed that the LRP protein was efficiently expressed in the endosperm of PA110.

**Fig. 4:**
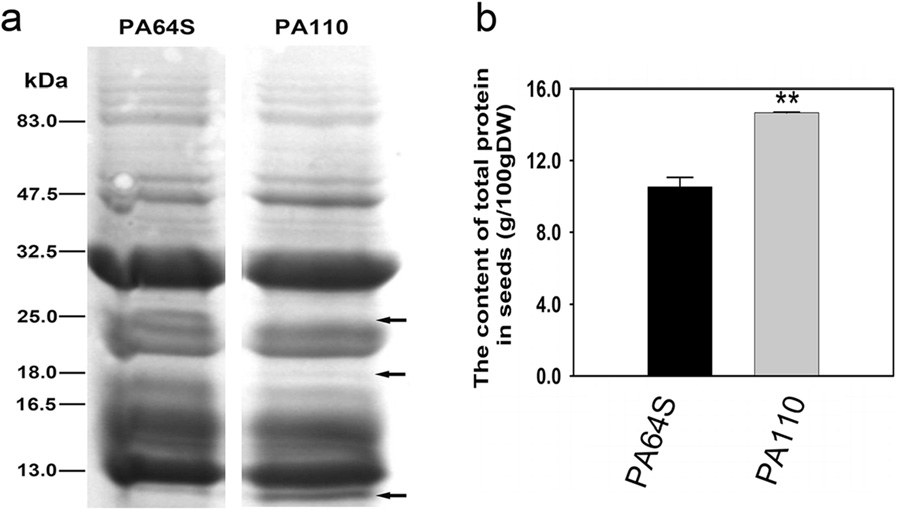
Effects of LRP on seed storage proteins and total proteins in the endosperm. **a** The accumulation of seed storage proteins in PA110 and wild-type by SDS-PAGE. Arrows indicate distinct bands between PA110 and wild-type. **b** The total protein content in the endosperm of PA110 and wild-type. *P* values were generated by the two-tailed *t* test, and **P < 0.01 denotes statistical significance

We also detected seed storage proteins (SSPs) in the mature endosperm of PA110 by SDS-PAGE analysis. Approximately same content of extracted proteins was loaded in each lane. The results revealed an extremely weak band with the expected size of the LRP protein, approximately 18 kDa (Fig. 4a). Furthermore, PA110 showed an additional approximately 10-kDa band but lacked an approximately 26-kDa band; the other bands differed little from wild-type (Fig. 4a). We further analyzed the total protein content in the endosperm of PA110, and found 14.7 % total protein for PA110, an increase of approximately 40 % compared with the 10.5 % for PA64S (Fig. 4b). These results indicated that the endosperm-specific expression of LRP provoked extensive synthesis of SSPs.

### PA110 and its hybrids significantly increases the content of Lys and other amino acids

**Fig. 5:**
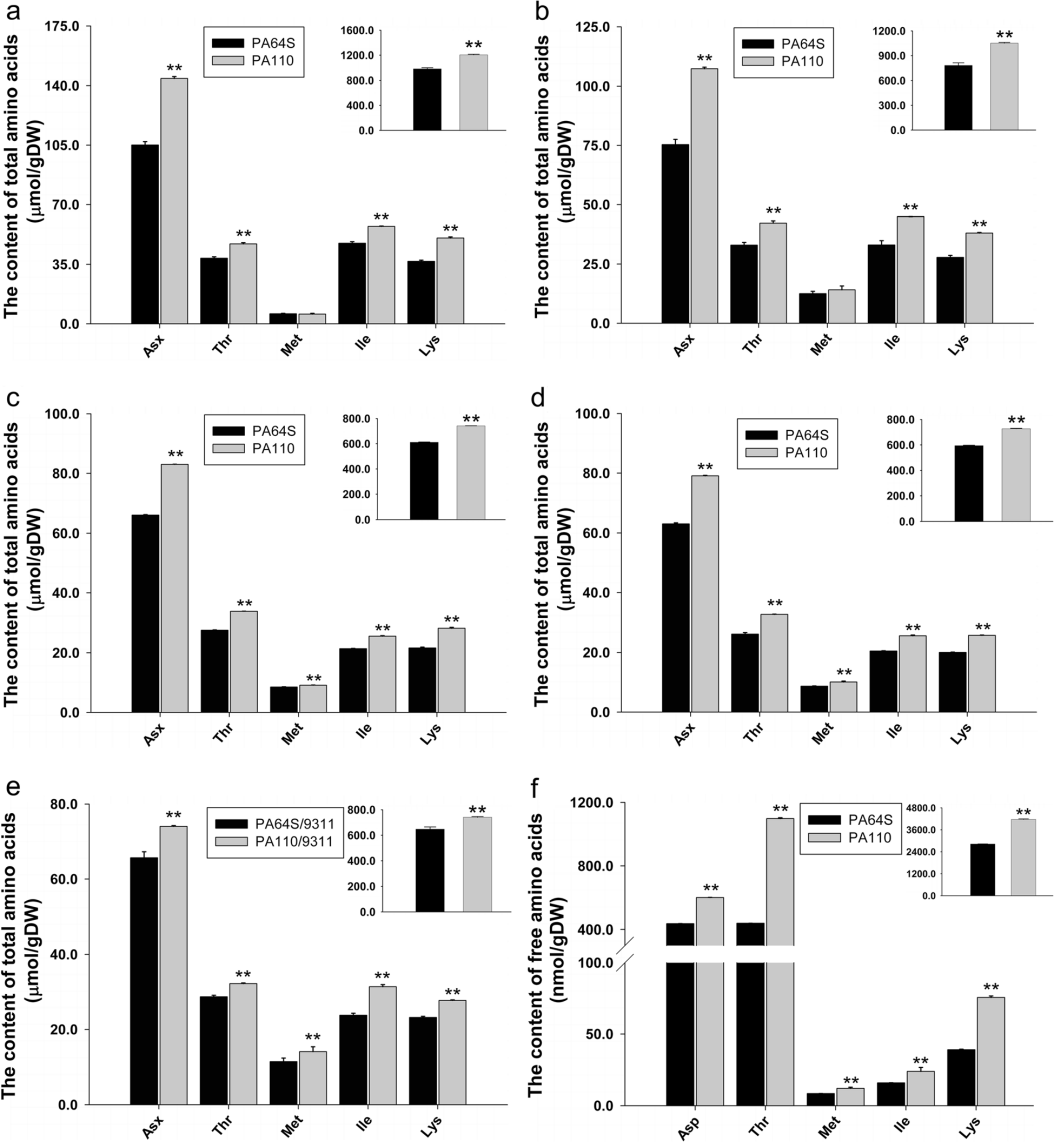
The content of amino acids involved in Lys metabolism in the seeds of different generations. **a** Total amino acid contents of brown rice in PA110 and wild-type PA64S in T_2_ generation in Hongkong, 2003. **b** Total amino acid contents of brown rice in PA110 and wild-type in T_8_ generation in Hangzhou, 2008. **c** Total amino acid contents of brown rice in PA110 and wild-type in T_14_ generation in Hangzhou, 2014. **d** The total amino acid content of PA110 and wild-type milling rice in the T_14_ generation. **e** The total amino acid content of PA110/9311 and PA64S/9311 brown rice in Hangzhou, 2014. **f** The free amino acid content of PA110 and wild-type brown rice in the T_14_ generation. Inserts indicate the total amount of all detectable amino acids. Gln and Asn were hydrolyzed to Glu and aspartate Asp under acidic conditions, and the final content of Glx was determined as the sum content of Gln and Glu; the final content of Asx was determined as the sum content of Asp and Asn. DW denotes dry weight. *P* values were generated by the two-tailed *t* test, and **P < 0.01 denotes statistical significance

To further determine whether the efficient expression of *LRP* in the endosperm of PA110 leads to an increase in Lys, we successively investigated the content of 16 amino acids in brown rice from PA110 and PA64S in three different generations. All results in PA110 seeds consistently showed significant increasing rates of the Lys content in a range from 30.14 to 37.24 %; the rate of average increase reached 34.76 % compared to the wild-type (Fig. 5a-c and Additional file 1: Table S1). The other 6 essential amino acids plus 9 non-essential amino acids detected also showed significant increases in all generations; exceptions were small decreases in methionine (Met), cysteine (Cys) and tyrosine (Tyr) in the T_2_ generation (Additional file 1: Table S1). Among the amino acids examined, the highest average increase for essential amino acids in the three measurements was found for isoleucine (Ile), which was increased by 25.40 %; the lowest was for Met, which increased an average of only 5.37 %. The highest average rate of increase for non-essential amino acids in the three measurements was found for histidine (His), which was increased by 28.38 %; the lowest was for Tyr, which increased an average of only 20.33 % (Additional file 1: Table S1). In addition, the relative content of Lys of the total amino acids increased an average of 3.61 % in PA64S to 3.86 % in PA110 (Additional file 1: Table S1). It is worth noting that Asp, threonine (Thr), Met and Ile, which are involved in the Asp metabolism pathway, showed significant increases in PA110 brown rice compared to wild-type, even though their biosynthesis competes for common substrates with that of Lys. These results indicated that the efficient expression of *LRP* in the endosperm of PA110 not only increases the content of Lys but also increases the content of other essential and non-essential amino acids, such that the total amount of amino acids increased by an average of 26.19 % compared with wild-type (Additional file 1: Table S1).

Similar to the results for brown rice, the contents of Lys and most amino acids in PA110 milling rice were increased from 16.41 to 28.02 %; only the Tyr content slightly decreased (0.24 %). The total amount of amino acids increased by 22.35 % compared with wild-type (Fig. 5d and Additional file 2: Table S2). The proportion of Lys among the total amino acids in PA110 milling rice was enhanced to 3.53 %, whereas that in PA64S was 3.37 % (Additional file 2: Table S2).

As PA110 is derived from the PTSMS line PA64S, we investigated the content of amino acids in the brown rice of hybrid PA110/9311 and the hybrid control PA64S/9311. Hybrid PA110/9311 exhibited a 19.60 % increase in the seed Lys content, and the other 6 detected essential amino acids also increased (Ile 32.00 %, Thr 12.21 %, phenylalanine (Phe) 13.06 %, leucine (Leu) 17.94 %, valine (Val) 21.09 %, Met 23.22 %), leading to a 14.34 % increase in the total amount compared with PA64S/9311 (Fig. 5e and Additional file 2: Table S2). The proportion of Lys in the total amino acid content in this transgenic hybrid accounted for 3.74 %, while only 3.58 % in the hybrid control (Additional file 2: Table S2). These results suggested that the endosperm-specific expression of *LRP* had similar effects on increasing the content of Lys and other amino acids in both the transgenic parent and its hybrid.

### The increase in amino acids by endosperm-specific expression of *LRP* is mainly due to protein-bound amino acids

The amino acids in the endosperm of rice consist of free and protein-bound amino acids. As the foreign LRP protein is rich in protein-bound Lys, endosperm-specific expression of *LRP* in PA110 was also assumed to increase the content of protein-bound Lys in seeds. To confirm this assumption, we measured the free amino acid content in brown rice of PA64S and PA110 and found that the free Lys content in PA110 increased 93.84 % compared to PA64S. The other free amino acids also showed an obvious increase, with a range from 7.33 to 150.40 %, which led to a 47.54 % increase in the total free amino acid content in PA110 compared with PA64S (Fig. 5f and Additional file 3: Table S3). Nevertheless, the content of free amino acids, including Lys and other amino acids, only accounted for approximately 1 % of the total amino acid content of both PA110 and PA64S (Additional file 4: Figure S1). Compared to the 26.19 % increase in the total amount in PA110 (Additional file 1: Table S1), the net increase in free amino acids was almost negligible, as observed in previous studies [6]. These results confirmed that the increase in amino acids in the PA110 endosperm was mainly caused by increases in protein-bound amino acids.

### Reduced amino acid catabolism leads to Lys accumulation in PA110 seeds

**Fig. 6:**
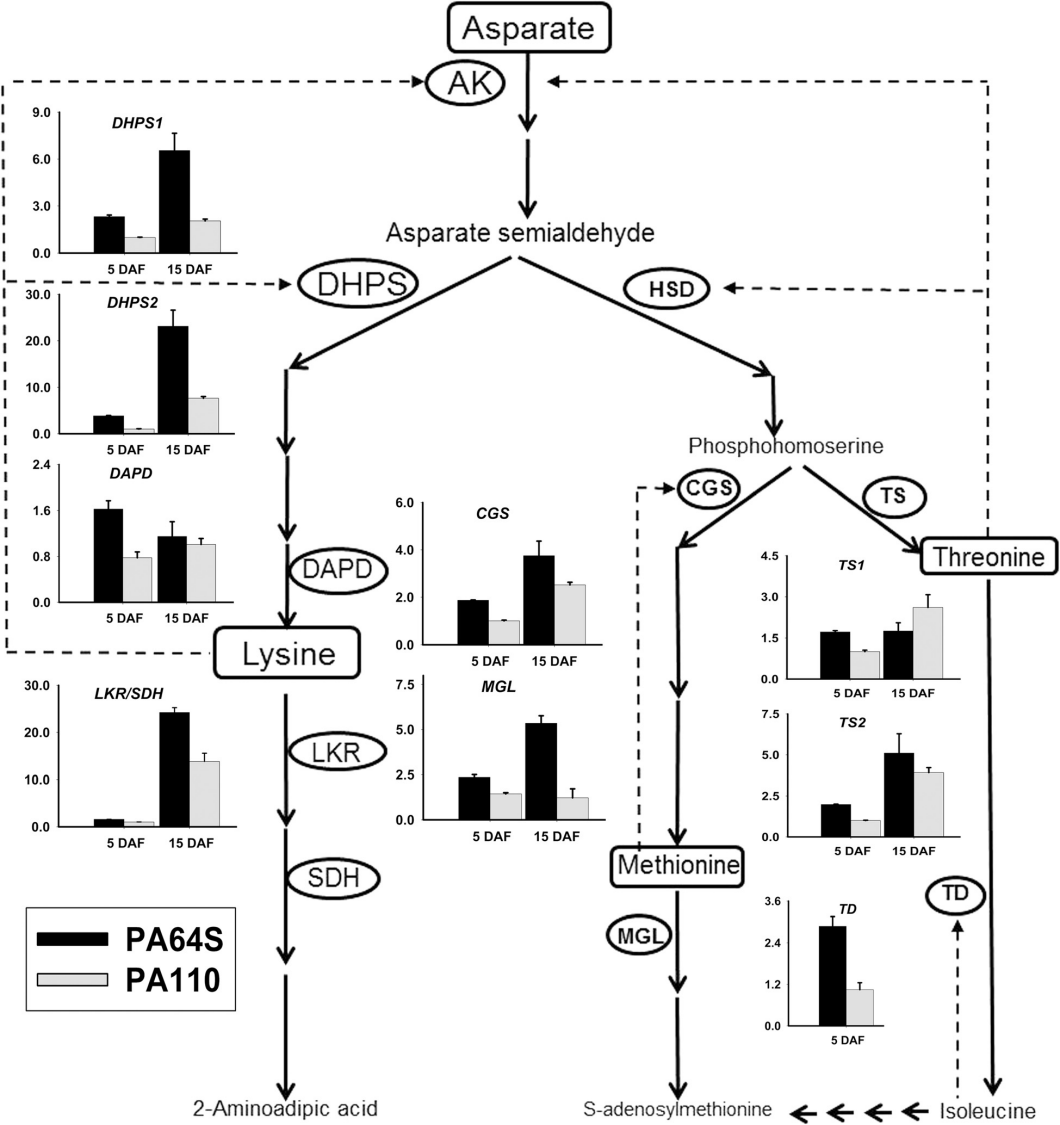
The aspartate family pathway leading to lysine, methionine and threonine. Key enzymes and amino acids in the pathway are specified. Inserts are the relative expression level of *OsDHPS*, *OsDAPD*, *OsLRK*/*SDH*, *OsCGS*, *OsMGL*, *OsTS*, and *OsTD* in PA110 and wild-type at 5 DAF and 15 DAF. Abbreviations: AK, aspartate kinase; DHPS, dihydrodipicolinate synthase; HSD, homoserine dehydrogenase; DAPD, diaminopimelate decarboxylase; LKR, Lys-ketoglutarate reductase; SDH, saccharopine dehydrogenase; CGS, cystathionine γ-synthase; TS, threonine synthase; MGL, methionine γ-lyase; TD, threonine dehydratase. Solid arrows indicate the flow of metabolic processes. Arrows with dashed lines indicate allosteric product feedback inhibition [Modified from [6, 18]]

To assess whether the increased synthesis and/or decreased catabolism of amino acids led to the observed increase in the total amino acid particularly Lys content in seeds, we monitored the expression levels of genes encoding key enzymes of Lys metabolism in developing seeds (Fig. 6). The results showed that the relative expression levels of most genes, regardless of whether they are involved in synthesis or catabolism, were lower in PA110 than in PA64S. The transcript abundance of the Lys biosynthetic *OsDAPD* gene in PA110 was nearly half of that in PA64S at 5 days after flowering (DAF). With the development of the endosperm, the expression of *OsDAPD* increased in PA110 yet decreased in PA64S, eventually approaching the same level as wild-type at 15 DAF (Fig. 6). Expression of the *OsLKR*/*SDH* gene, which encodes a bifunctional LKR and SDH enzyme in the Lys catabolic pathway, was roughly the same between PA110 and PA64S at the 5 DAF stage and was approximately 16-fold in PA64S but only approximately 13-fold in PA110 at the 15 DAF stage. These results demonstrated obviously lower expression of *OsLKR*/*SDH* in PA110 compared to PA64S (Fig. 6). These expression patterns of biosynthetic *OsDAPD* and catabolic *OsLKR*/*SDH* between PA110 and PA64S suggested that reduced Lys catabolism might account for the accumulation of Lys in the seeds of PA110.

Because Met competes with Lys in the Asp pathway [2, 9, 11, 13], we detected the expression of *OsCGS*, involved in Met biosynthesis, and *OsMGL*, involved in Met catabolism. The expression level of *OsCGS* in PA110 was significantly lower compared to that in PA64S at 5 DAF (Fig. 6). As the endosperm develops, the expression of *OsCGS* increased in both PA110 and PA64S, with the difference between PA110 and PA64S narrowing from approximately 1.9-fold at 5 DAF to approximately 1.4-fold at 15 DAF. The expression level of *OsMGL* in PA64S was significantly higher than that in PA110 at the 5 DAF stage, strongly increasing in PA64S endosperm but with no obvious changes from 5 DAF to 15 DAF in PA110 (Fig. 6). These results prove that the higher accumulation of Met in transgenic plants might be attributed to the lower Met catabolism in PA110 compared to PA64S.

The *OsTS* gene, which has two homologs, is responsible for the biosynthesis of Thr, whereas the *OsTD* gene is involved in Thr catabolism. At 5 DAF, the expression level of *OsTS1* was lower in the endosperm of PA110 than in PA64S; during grain filling, the expression of *OsTS1* sharply increased in the PA110 endosperm, though no obvious changes were observed in PA64S. Thus, the transcript abundance of *OsTS1* in PA110 was even greater than that in PA64S at the 15 DAF stage (Fig. 6). *OsTS2* demonstrated similar expression patterns as *OsCGS*. The *OsTD* expression level in 5 DAF endosperm of PA110 was significantly lower than that of wild-type, but no expression were detected in the 15 DAF endosperm of both PA110 and wild-type (Fig. 6). All of these results suggested that the enrichment of amino acids in PA110, which enabled the extensive synthesis of LRP and SSPs, may largely result from the reduced catabolism of amino acids.

### Agronomic performance of PA110 and its hybrid

**Fig. 7:**
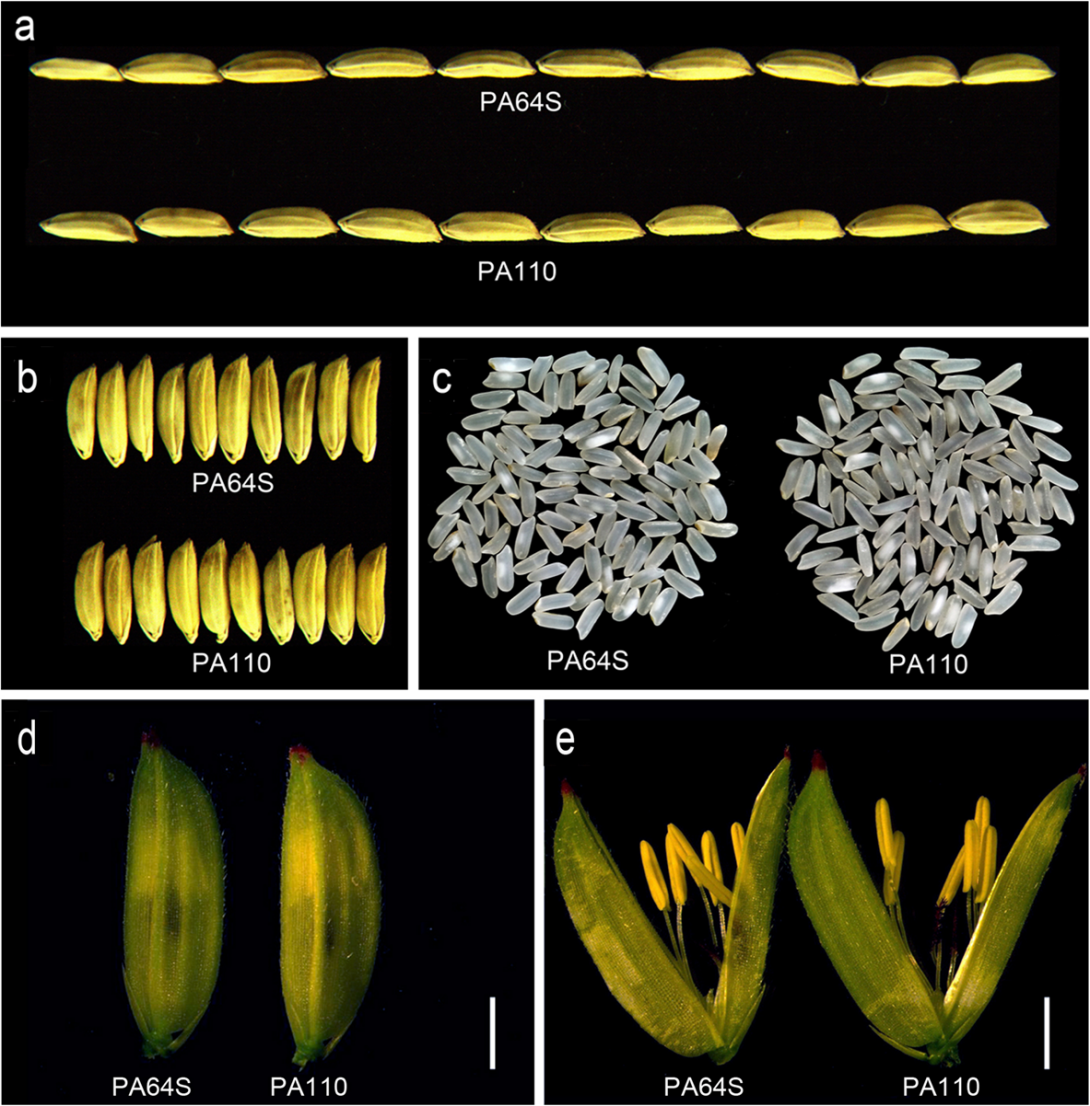
Morphology of the flower organ, grain size and chalkiness of PA110 and PA64S. **a** Grain length. **b** Grain width. **c** Grain chalkiness. **d**-**e** Flower morphology. Scale bars, 2 mm

As listed in Table 1, PA64S exhibited a heading date of 87 days, 7.0 tillers per plant, 18.6 g of 1000-grain weight, a 21.7 % chalkiness rate, 8.5 mm for grain length, 2.7 mm for grain width, and a seed germination rate of 90.3 %. In comparison with PA64S, PA110 exhibited normal plant growth and development for most traits, except that the heading date was delayed to 91 days, the 1000-grain weight declined to 15.7 g, and the seed germination rate decreased to 74.3 % (Fig. 7; Table 1). Because PA110 is derived from PA64S, a male sterile line of a two-line hybrid, we examined fertility-related traits such as pollen fertility under both sterile and fertile conditions and the sperm and vegetative nucleus status under the fertile condition. The results showed no obvious changes in pollen fertility between PA110 and PA64S (Additional file 5: Figure S2). Agronomic traits were also examined in the hybrids of PA110 and wild-type PA64S. Although obvious negative effects on some agronomic traits were observed in the parent line PA110 (Table 1), its hybrid PA110/9311 illustrated little differences from the hybrid control PA64S/9311 with regard to most agronomic traits, especially seed germination and 1000-grain weight (Additional file 6: Table S4).

**Table 1:** Agronomic performance of PA110 and wild-type PA64S (Sanya, 2014)

Line	Heading date (days)	No. of tillers per plant	1000-grain weight (g)	Chalkiness rate (%)	Grain length (mm)	Grain width (mm)	Seed germination rate (%)
PA64S (CK)	87	7.0 ± 0.3	18.6 ± 0.2	21.7 ± 1.5	8.5 ± 0.0	2.7 ± 0.0	90.3 ± 0.9
^a^PA110	91	7.2 ± 0.2	15.7 ± 0.1**	22.7 ± 1.8	8.5 ± 0.0	2.7 ± 0.0	74.3 ± 1.2**

^a^Significant differences are at the levels of **P < 0.01

## Discussion

Here, we report the development of high Lys rice via introducing a winged been lysine rich protein gene under the control of rice GT1 promoter into an elite rice photoperiod-thermo-sensitive male sterility cultivar Peiai64S. The obtained line and its derivative hybrid showed significant increase in Lys and many other essential and non-essential amino acid contents (Fig. 5; Additional file 1: Table S1; Additional file 2: Table S2). Correspondingly, the total seed protein content was also heightened by 40 % (Fig. 4b). These results were consistent with the previous reports. The total seed protein content in the transgenic maize harboring a lysine-rich protein gene *sb401* was heightened by 11.6 to 39.0 % [30]. The similar rates of increase in both amino acids and total seed protein were also observed in seed-specific expression of a cotton-sourced *GhLRP* gene and a potato-sourced *SBgLR* gene in maize [15, 31]. The above instances together with our results hence demonstrate that the endosperm-specific expression of lysine-rich proteins could provoke extensive synthesis of SSPs and thus led to increase in Lys and total amino acid contents.

In this study, the Lys content of the endosperm consistently remained at a high level, with an average 34.76 % increase in Lys in three different generations compared to the wild-type PA64S. These results indicated that the elevation in Lys and total amino acids in PA110 were due to the transgenic *LRP* gene and were stably heritable; thus, the *LRP* gene might have value in the improvement of the Lys content of grain (Fig. 5 and Additional file 1: Table S1). Previous studies showed that Lys increases in crop seeds are usually accompanied by the enrichment of Thr and Gly and a decrease in Leu and Glu [12, 27]. However, these 4 amino acids all increased in the three generations in our study, indicating the feasibility of simultaneously increasing these 4 amino acids in seeds (Additional file 1: Table S1). Furthermore, in addition to Lys, most of the essential amino acids showed significant increases in PA110 in all three generations, which suggested that the *LRP* gene may be valuable for improving the levels of all essential amino acids in seeds.

To enhance the level of free Lys in Arabidopsis seeds, Zhu and Galili [33] introduced a bacterial feed-back-insensitive *DHPS* gene under the control of seed-specific promoter in an Arabidopsis knockout mutant lacking a bifunctional *LKR*/*SDH*. The resulted transgenic plants showed a nearly 80-fold increase in the level of seed free Lys. Since the Lys are known to toxic to plants [10], this dramatic increase in seed free Lys caused significant retardation on seed germination and the lowest seed germination rate observed was less than 5 % [33]. Such severe retardation effect on seed germination was also observed in many other studies [4, 16, 22]. However, in the case of this study, the decrease in seed germination rate in PA110 happened to a mild degree with a rate less than 16 %. This might be due to a low level of increase in free seed Lys in PA110 comparing with that in wild-type (Fig. 5f and Additional file 3: Table S3). These findings indicate that the genetic engineering approach by expressing genes that encode quality proteins with balanced Lys composition may be more preferable for grain nutrient improvement.

We also observed that 1000-grain weight in PA110 was unexpectedly affected by the expression of *LRP* gene, and showed differences from those in wild-type PA64S (Table 1). Furthermore, in our other experiment, we have introduced the transgenic locus inclusion of *LRP* gene into the elite rice line 9311 through 3-generations of backcrossing, and we also observed that the resultant line (9311 genetic background with *LRP* gene) had 26.3 g of 1000-grain weight averagely, which were significantly different from those 28.2 g of 1000-grain weight in wild-type 9311 (unpublished data). Peng et al. [21] found that the accumulation of protein in the seeds of transgenic plants resulted in a significant decrease in the starch content. Similarly, we also found 40 % increase of total seed protein content accompanying with 10 % decrease of total seed starch content in PA110 (Fig. 4b; Additional file 7: Figure S3). The starch content is the major component of rice and accounts for about 90 % of dry matter [5]. As shown in Fig 4b, the total seed protein detected in Peiai64S accounts for 10 % of its dry weight. So that in PA110 the net increase in total seed protein could not compensate the net decrease in total seed starch and the final performance is the reduction in 1000-grain weight (Table 1).

Previous efforts to improve Lys content focused only on the increase of Lys content in the target transgenic plants including rice, and paid little attention to its enhancement in their hybrid seeds. China is the main producer of hybrid rice and has made significant progress in hybrid rice breeding [17]. The hybrid PA64S/9311 was one of the most widely cultivated hybrids, accounted for 7 million ha of the total planting area in China by 2007 [35]. In our study, we examined the amino acids content in the transgenic hybrid and found that the PA110/9311 exhibited a substantial increase (19.6 %) in the content of Lys and other amino acids compared to PA64/9311, suggesting that the heterozygous genotype of the *LRP* gene could also be used for Lys improvement in rice via *LRP* transgenic engineering (Additional file 2: Table S2). Further, some agronomic traits in PA110 were unexpectedly affected by the expression of *LRP* gene, but we noted that most agronomic traits showed little differences between PA110/9311 and PA64S/9311 (Additional file 7: Figure S3; Additional file 6: Table S4), and the negative effects, including decreased 1000-grain weight and seed germination rate occurred in PA110 were not observed in PA110/9311.

As shown in Additional file 1: Table S1, the Lys content in PA110 seeds increased more than 30 % compared to the wild-type, while in its hybrid PA110/9311 only increased approximate 20 % compared to hybrid control (Additional file 2: Table S2). Previous reports showed that over accumulation of Lys in seeds caused abnormal grain development [16, 25]. In this study, while over accumulation of Lys content in PA110 seeds caused some negative effects in its agronomic traits, moderate increase of Lys in hybrid PA110/9311 seeds appeared to avoid the undesirable agronomic traits. Based on our results, we propose a strategy for improving the Lys level in the seeds of hybrid rice: To use male sterile lines as a transgenic receptor to moderately express a protein with ample or balanced Lys to enhance the Lys content in seeds.

In the endosperm of PA110, *OsDAPD* involves in Lys biosynthesis, while *OsLKR*/*SDH* in Lys catabolism, and *OsDHPS* in the upstream regulation of Lys biosynthesis showed significantly altered expression patterns compared to wild-type (Fig. 6). *OsDAPD* presented an upward trend of expression from 5 DAF to 15 DAF in the PA110 endosperm; the reverse was true for PA64S, which might have caused the higher accumulation of Lys in PA110. *OsLKR*/*SDH* showed lower expression levels in the endosperm of PA110 at 15 DAF, suggesting reduced Lys catabolism. Both of these factors might contribute to the increase in Lys in the seeds of PA110 compared to wild-type (Fig. 6). *OsDHPS1* and *OsDHPS2*, two key upstream genes in the Lys biosynthetic pathway coincided expression pattern in that they increased notably with grain filling in both PA110 and PA64S, though the increase in PA110 was to a lesser extent than in PA64S (Fig. 6). This indicated that the accumulated Lys in PA110 had a feedback inhibition effect on DHPS, consistent with previous studies showing that the over-accumulation of Lys would down-regulate the upstream gene in Lys biosynthesis, *DHPS*, via feedback inhibition [1, 14, 16]. The content of Thr in the brown rice of PA110 increased by approximately 24 % compared to PA64S (Fig. 5; Additional file 1: Table S1). The expression of *OsTS1* in PA110 was significantly elevated from 5 DAF to 15 DAF but only subtly altered in wild-type, and the expression of *OsTS2* increased with grain filling in the endosperm of both PA110 and wild-type (Fig. 6), suggesting that *OsTS1* rather than *OsTS2* is possibly the causal gene encoding the TS enzyme in rice. Because Lys originates from Asp, we also detected the expression of *OsAK* and *OsHSD*, both of which are involved in Asp catabolism, and found significantly different expression levels for *OsAK1*, *OsAK2*, *OsAK3* and *OsHSD* between PA110 and the wild-type PA64S (Additional file 8: Figure S4). Overall, the above results indicated that expression of the exogenous *LRP* gene in the seeds of transgenic plants remarkably influenced Lys metabolism in PA110 and that changes in the expression of metabolism-related genes, especially the catabolism governing genes, might have led to increases in Lys and other amino acids.

Furthermore, some differences in SSPs were observed between PA110 and the wild-type PA64S by SDS-PAGE (Fig. 4a). Therefore, we examined the transcript levels of genes involved in grain protein biosynthesis in the endosperm. Two homologs, O*sGlobulin1* and *OsGlobulin2*, showed similar expression pattern, with the transcripts dramatically increased during grain filling in both PA110 and wild-type. However, the increase in PA110 (~43-fold for *OsGlobulin1* and ~3-fold for *OsGlobulin2*) was much lower than that in PA64S (~384-fold for *OsGlobulin1* and ~64-fold for *OsGlobulin2*) from 5 DAF to 15 DAF (Additional file 9: Figure S5a and b). This expression pattern was in agreement with the SDS-PAGE results that one band at ~26 kDa (globulin) was not present in PA110 (Fig. 4a). *OsGluA2*, *OsGluA3*, and *OsGluB1* exhibited expression patterns similar to *OsGlobulin1* in PA110 and wild-type (Additional file 9: Figure S5c-e). *OsGluB4* expression increased with grain filling in PA110, whereas it decreased in PA64S (Additional file 9: Figure S5f). The transcript abundance of *OsProlamin* also increased with grain filling in PA110 and wild-type, but the increase was much higher in PA110 than in PA64S (Additional file 9: Figure S5g), indicating that increased *OsProlamin* expression might account for the extra band at 13 kDa in PA110. These results strongly suggest that the expression of foreign *LRP* in the endosperm had significant effects on the expression of a large portion of genes participating in SSP biosynthesis in developing seeds.

## Conclusions

Lys is considered to be the first limiting essential amino acid in rice. The present results show that endosperm-specific expression of foreign *LRP* gene significantly increased the Lys content in the seeds of both transgenic plant and its hybrids. Persistent investigation of amino acids also indicates that the Lys increase is stably heritable. The transcript abundance of genes involving Lys metabolism revealed that the reduced amino acid catabolism led to the accumulation of Lys and other amino acids in the transgenic plants. The hybrid of transgenic plant displayed little negative effects on grain, and the result also suggested that expression of *LRP* in seeds may have promising applications in improving Lys levels in hybrid rice.

## Methods

### Plant materials and agronomic traits

The elite rice photoperiod-thermo-sensitive male sterility cultivar *Oryza sativa* ssp. *Indica* cv. Peiai64S, from China National Hybrid Rice R&D Center, was used as a transgenic recipient. Transgenic plants from the T_0_ to T_3_ generations were planted in the greenhouse at the Chinese University of Hong Kong (2001–2003). The transgenic plants of the ensuing generations were planted in paddy fields in Hangzhou in summer to maintain self-sterility and in Sanya in winter to maintain self-fertility (2004–2014). The elite rice cultivar *Oryza sativa* ssp. *Indica* cv. 9311, from Agricultural Research Institute of Lixiahe, China, was used to cross with PA110 and PA64S to obtain the hybrids PA110/9311 and PA64S/9311, respectively.

Other relevant materials were planted in Hangzhou for the evaluation of agronomic traits. A randomized block design was adopted with three replications. Each plot contained 6 rows, with 12 plants per row and a spacing of 17 cm between plants and 26 cm between rows. The plants in the middle of the row in each plot were investigated for agronomic traits, including heading date, tillers per plant, number of grains per panicle, spikelet fertility, 1000-grain weight, seed germination and yield per plant. Grain quality traits, such as chalkiness and grain size, were also investigated in PA110 and PA64S. The data were collected from 5 plants, the difference between samples are examined by the *t* test. All tissue samples were harvested at approximately the seedling, tillering, and grain-filling stages (5 DAF, 15 DAF, and mature), immediately frozen in liquid nitrogen, and stored at −80 °C for the measurement of amino acids. All field works carried out in this study comply with institutional, national, and international guidelines and conforms to the Regulations on Administration of Agricultural Genetically Modified Organisms Safety, China.

Starch content was measured from mature seeds using K-TSTA (Megazyme, Ireland), and the detailed procedure was according to the manual.

### Vector construction and rice transformation

*LRP* cDNA was isolated by reverse-transcription PCR using total mRNA from developing seeds of *Psophocarpus tetragonolobus* (L.) DC [23], and (5′-CGA*GGATCC*ATGGGTGTTTTCACATATG-3′) and (5′-GAC*GAGCTC*TTCAATTGTATTCAGGATGG-3′) were used as forward and reverse primers, respectively. The primers 5′-GGC*AAGCTT*CACCCTCAATATTTGGAA-3′ and 5′-CGA*GGATCC*GTTGTTGTAGGACTAATGAA-3′ were used for amplifying the GT1 promoter from the elite *indica* cultivar Zhenshan 97. Three restriction enzymes, *Hin*dIII, *Bam*HI and *Sac*I, were used to clone the rice GT1 promoter (Accession No. D00584) and *LRP* gene into the binary vector pSB130. The *HPT* gene was utilized as the selection marker, with primers 5′-GCTGTTATGCGGCCATTGTC-3′ and 5′-GACGTCTGTCGAGAAGTTTC-3′ used for PCR analysis. Rice calli induced from PA64S mature embryos were used for *Agrobacterium*-mediated transformation, performed using a previously described procedure [29].

### Transgene copy number detection

Transgene copy numbers were detected by Southern blot hybridization analysis. Approximately 12 μg of total DNA was digested and blotted onto nylon membranes (GE Healthcare, UK). A 477-bp *LRP* gene fragment was amplified using 5′-ATGGGTGTTTTCACATATG-3′ and 5′-TCAATTGTATTCAGGATGG-3′ to generate the hybridization probe by DIG DNA Labeling Mix (Roche, Switzerland). Detection was performed using a DIG Luminescent Detection Kit for Nucleic Acids (Roche, Switzerland). The detailed procedure was according to the manual.

### qRT-PCR analysis

Total RNA was isolated from leaves using TRIzolRNA (Invitrogen, Carlsbad, USA) and from seeds using RNeasy Plant Mini Kit (QIAGEN, Germany) at the early grain-filling stage (5 DAF), milky stage (15 DAF), and mature stage. Each sample consisted of mixed RNA extracted from more than 20 plants. First-strand cDNA was generated using the Perfect Real Time Primescript RT reagent (TaKaRa, Japan); the details followed the kit manual. qRT-PCR was performed using a Roche LightCycler® 96 system with three technical replicates, as described by Zhang et al. [32]. The rice *actin* gene was used for an internal control to assay the relative expression levels of *AK1*, *AK2*, *AK3*, *CGS*, *DHPS1*, *DHPS2*, *HSD1*, *HSD2*, *LKR/SDH*, *TS1*, *TS2*, *DAPD*, *MGL*, *TD*, *GluB1*, *GluA2*, *GluA3*, *GluB4*, *Globulin1*, *Globulin2* and *Prolamin*. The gene-specific primers used for qRT-PCR are listed in Additional file 10: Table S5.

### Amino acid analysis

For the analysis of amino acids, samples of the T_2_, T_8_, and T_14_ generations were harvested in 2003 (Hong Kong), 2008 (Hangzhou) and 2014 (Hangzhou), respectively. All samples were placed in liquid nitrogen and ground into powder. Each sample consisted of a mixed powder prepared from more than 500 grains. Total amino acids (Asp, Thr, Glu, Gly, Ala, Val, Met, Ile, Leu, Tyr, Phe, His, Arg, Ser, Cys and Lys) in the seeds were treated with acid hydrolysis. Approximately 0.2 g seed flour was suspended in 5 mL 6 mol/L HCl under nitrogen conditions and heated at 110 °C for 24, and the HCl was then evaporated in a rotary evaporator at 60 °C for 15 min. The residues were dissolved in 10 mL 0.02 mol/L HCl and filtered through a 0.45-μm Nylon Acrodise filter. Free amino acids were extracted from 200 mg of bulked rice flour or fresh leaves using 2 mL of 3 % (*w/v*) sulfosalicylic acid dehydrate at 4 °C for 2 h. Extracts were centrifuged at 15000 rpm and 4 °C for 5 min to remove debris, and the supernatants were filtered through a 0.45-μm Nylon Acrodise filter. The samples were separated into 3 parts for technical replicates and assayed using an automatic amino acid analyzer (L-8900, Hitachi, Japan).

### Seed protein extraction and Western blot analysis

Total protein was extracted from 1 mg mature seed powder in buffer (125 mmol/L Tris–HCl, pH 6.8, 4 mol/L Urea, 4 % SDS, 5 % mercaptoethanol), as previously described [28]. The content of extracted proteins for SDS-PAGE was analyzed using Bradford methods [3]. The LRP protein purified from the seeds of *P. tetragonolobus* (L.) DC was used as the quantitative reference. Protein samples were separated by 12 % SDS/PAGE and transferred onto a nitrocellulose membrane; rabbit immune serum diluted 1:10000 was used in the Western blot assay. Detection was according to the procedures in the Western blot chemiluminescent detection system manual (Millipore, USA). Seed protein content analysis was based on the method of Chinese National Food Safety Standard Determination of protein in foods (GB 5009.5-2010).

## Abbreviations

AK, aspartate kinase; Asp, aspartate; CGS, cystathionine γ-synthase; DAF, days after flowering; DAPD, diaminopimelate decarboxylase; DHPS, dihydrodipicolinate synthase; GT1, glutelin1; HPT, hygromycin phosphotransferase; HSD, homoserine dehydrogenase; LKR/SDH, lysine-ketoglutarate reductase/saccharopine dehydrogenase; LRP, lysine-rich protein; Lys, lysine; MGL, methionine γ-lyase; PTSMS, photoperiod-thermo-sensitive male sterility; qRT-PCR, quantitative real time-PCR; SSP, seed storage protein; TD, threonine dehydratase; TS, threonine synthase.

## Additional files

Additional file 1: Table S1. Total amino acid content in brown rice of PA110 and wild-type in different generations. (XLSX 13 kb) Additional file 2: Table S2. Total amino acid content in milling rice of PA110 and brown rice of its hybrid (Hangzhou, 2014). (XLSX 12 kb) Additional file 3: Table S3. Free amino acid content in brown rice of PA110 and wild-type (Hangzhou, 2014). (XLSX 11 kb) Additional file 4: Figure S1. Amino acid content in T14 generation of PA110 and PA64S. (JPG 1752 kb) Additional file 5: Figure S2. Pollen fertility of PA110 and wild-type. (**a**) and (**b**) Pollen under the sterile condition. (**c**) and (**d**) Pollen under the fertile condition. Matured pollen was stained with 1 % potassium iodide, dark staining indicates viable pollen. Scale bars, 50 μm. (**e**) and (**f**) Fluorescent staining by 4′,6-diamidino-2-phenylindole (DAPI, 1 μg/mL) detected the nucleus status of pollen under fertile condition, light and faint staining indicate two sperm nucleus and vegetative nucleus, respectively. Scale bars, 5 μm. (JPG 5437 kb) Additional file 6: Table S4. Agronomic performance of PA110 hybrid (Hangzhou, 2014). (XLSX 10 kb) Additional file 7: Figure S3. Total starch content in seeds of PA110 and its hybrid. Error bar indicates s.e.m. calculated from 3 technical replicates. Columns with the same letters are not significantly different at *P* < 0.01. (JPG 1437 kb) Additional file 8: Figure S4. qRT-PCR analysis of rice genes involved in Asp metabolism in seeds. Error bar indicates s.e.m. calculated from 3 technical replicates. (JPG 3773 kb) Additional file 9: Figure S5. qRT-PCR analysis of rice genes involved in grain protein biosynthesis. Error bar indicates s.e.m. calculated from 3 technical replicates. (JPG 610 kb) Additional file 10: Table S5. Primer pairs corresponding to qRT-PCR of genes involving in Asp metabolism and grain storage protein. (XLSX 11 kb)
