# Supplementary figures and images for: Development of high-lysine rice via endosperm-specific expression of a foreign LYSINE RICH PROTEIN gene
Source: BMC Plant Biol. 2016 Jun 29;16:147. doi: 10.1186/s12870-016-0837-x (PMC4928333; doi:10.1186/s12870-016-0837-x)

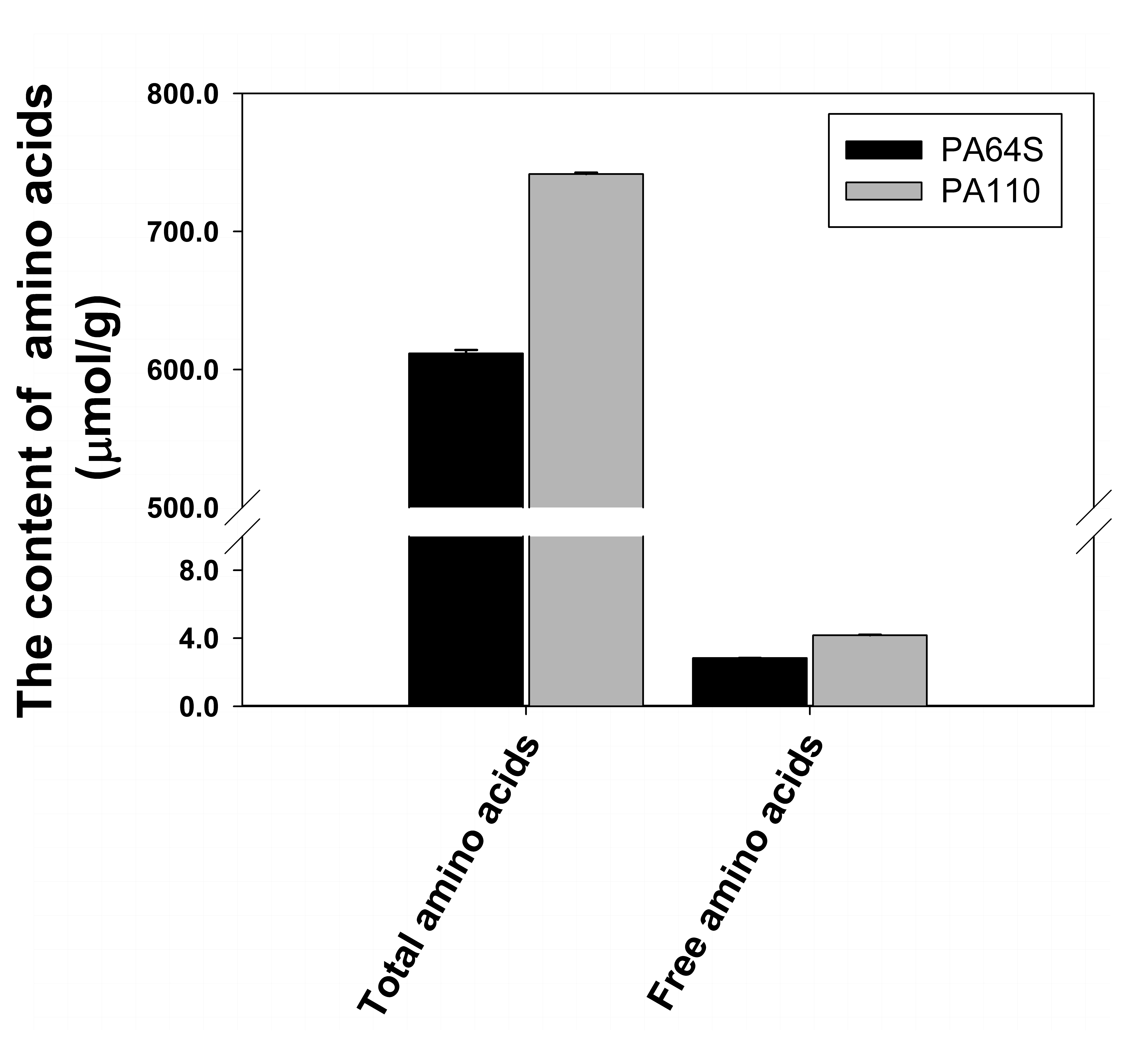

Supplement: Additional file 4: Figure S1. — Amino acid content in T14 generation of PA110 and PA64S. (JPG 1752 kb) [file 12870_2016_837_MOESM4_ESM.jpg]

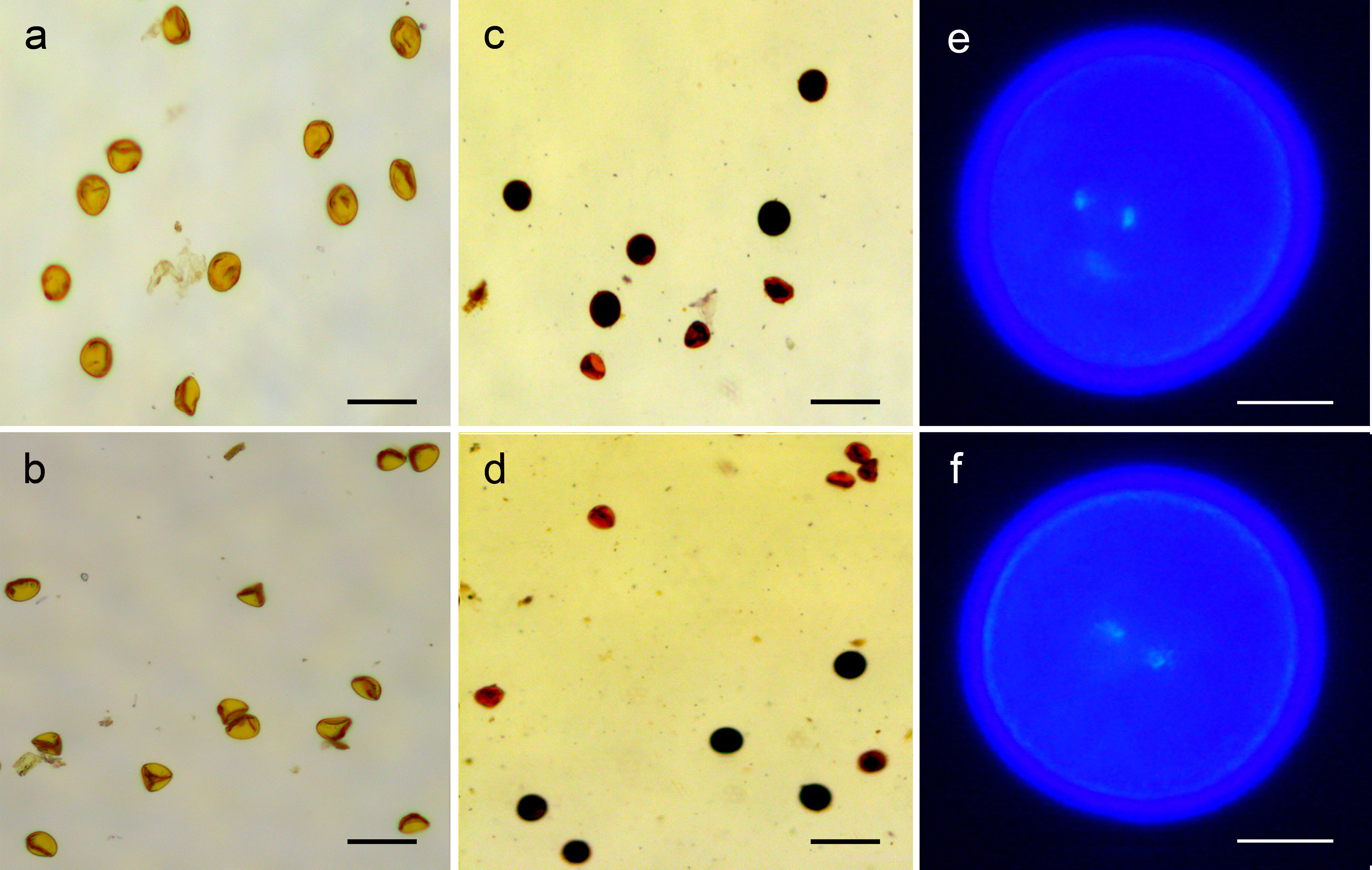

Supplement: Additional file 5: Figure S2. — Pollen fertility of PA110 and wild-type. (a) and (b) Pollen under the sterile condition. (c) and (d) Pollen under the fertile condition. Matured pollen was stained with 1 % potassium iodide, dark staining indicates viable pollen. Scale bars, 50 μm. (e) and (f) Fluorescent staining by 4′,6-diamidino-2-phenylindole (DAPI, 1 μg/mL) detected the nucleus status of pollen under fertile condition, light and faint staining indicate two sperm nucleus and vegetative nucleus, respectively. Scale bars, 5 μm. (JPG 5437 kb) [file 12870_2016_837_MOESM5_ESM.jpg]

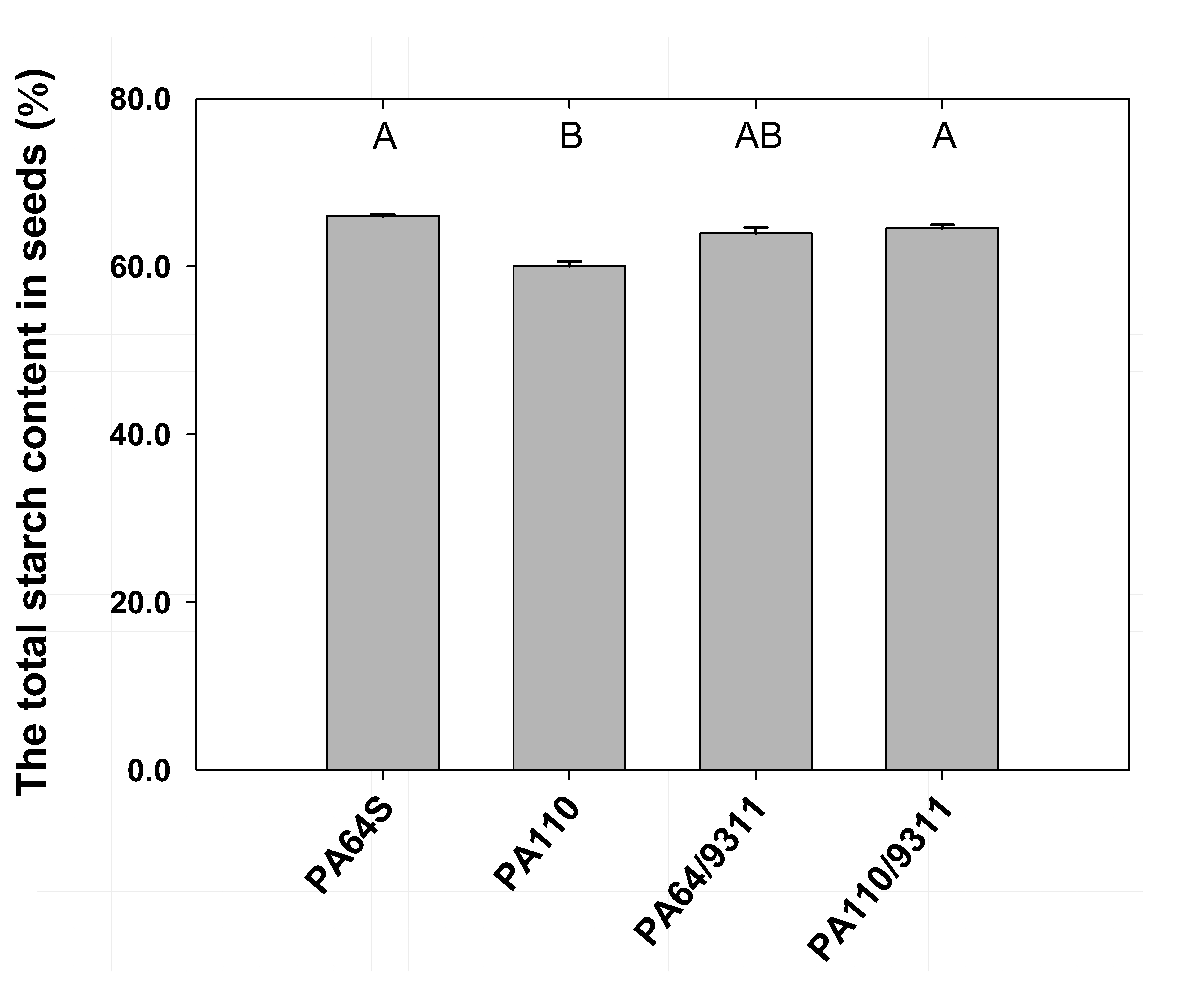

Supplement: Additional file 7: Figure S3. — Total starch content in seeds of PA110 and its hybrid. Error bar indicates s.e.m. calculated from 3 technical replicates. Columns with the same letters are not significantly different at P < 0.01. (JPG 1437 kb) [file 12870_2016_837_MOESM7_ESM.jpg]

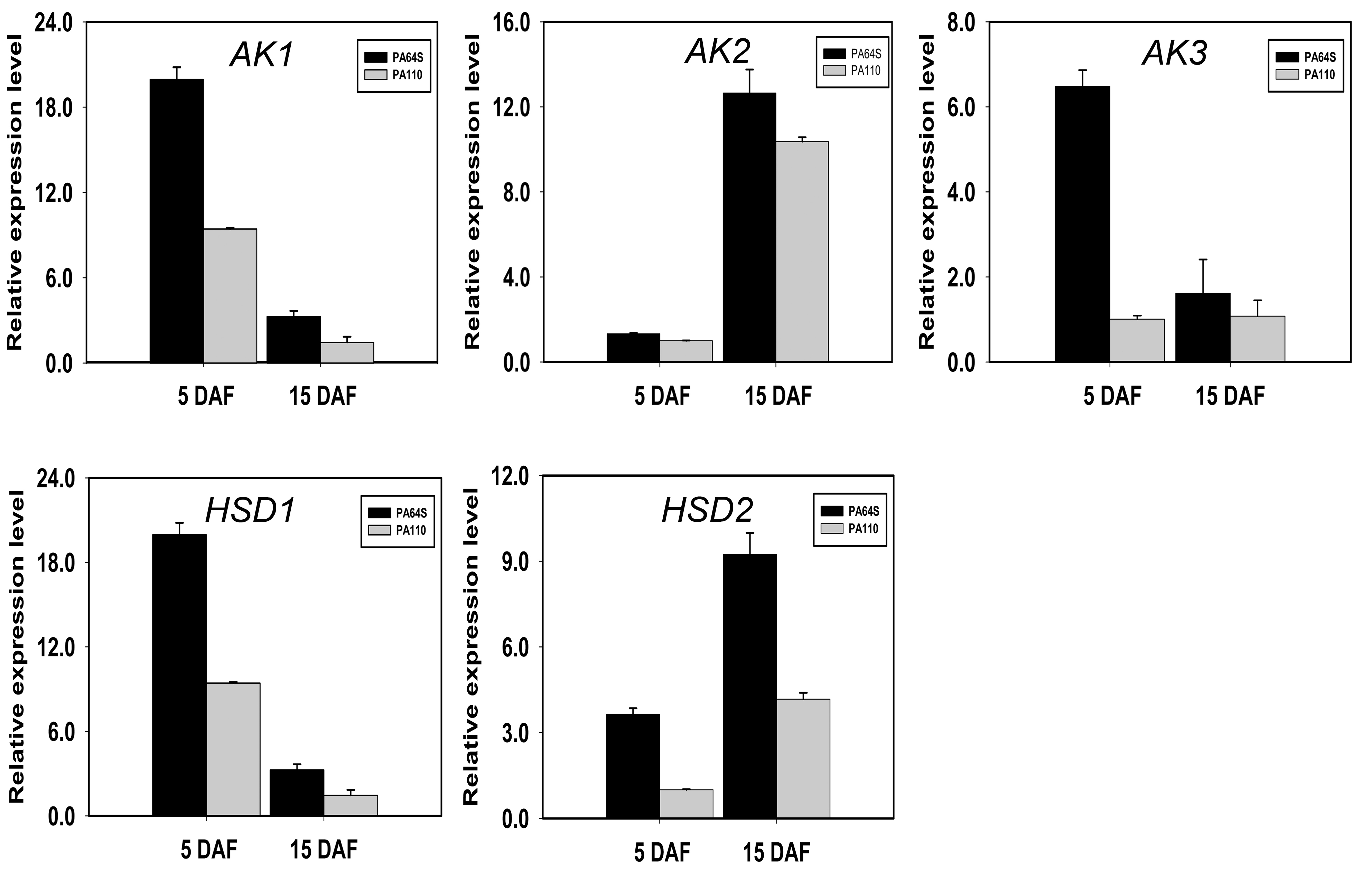

Supplement: Additional file 8: Figure S4. — qRT-PCR analysis of rice genes involved in Asp metabolism in seeds. Error bar indicates s.e.m. calculated from 3 technical replicates. (JPG 3773 kb) [file 12870_2016_837_MOESM8_ESM.jpg]

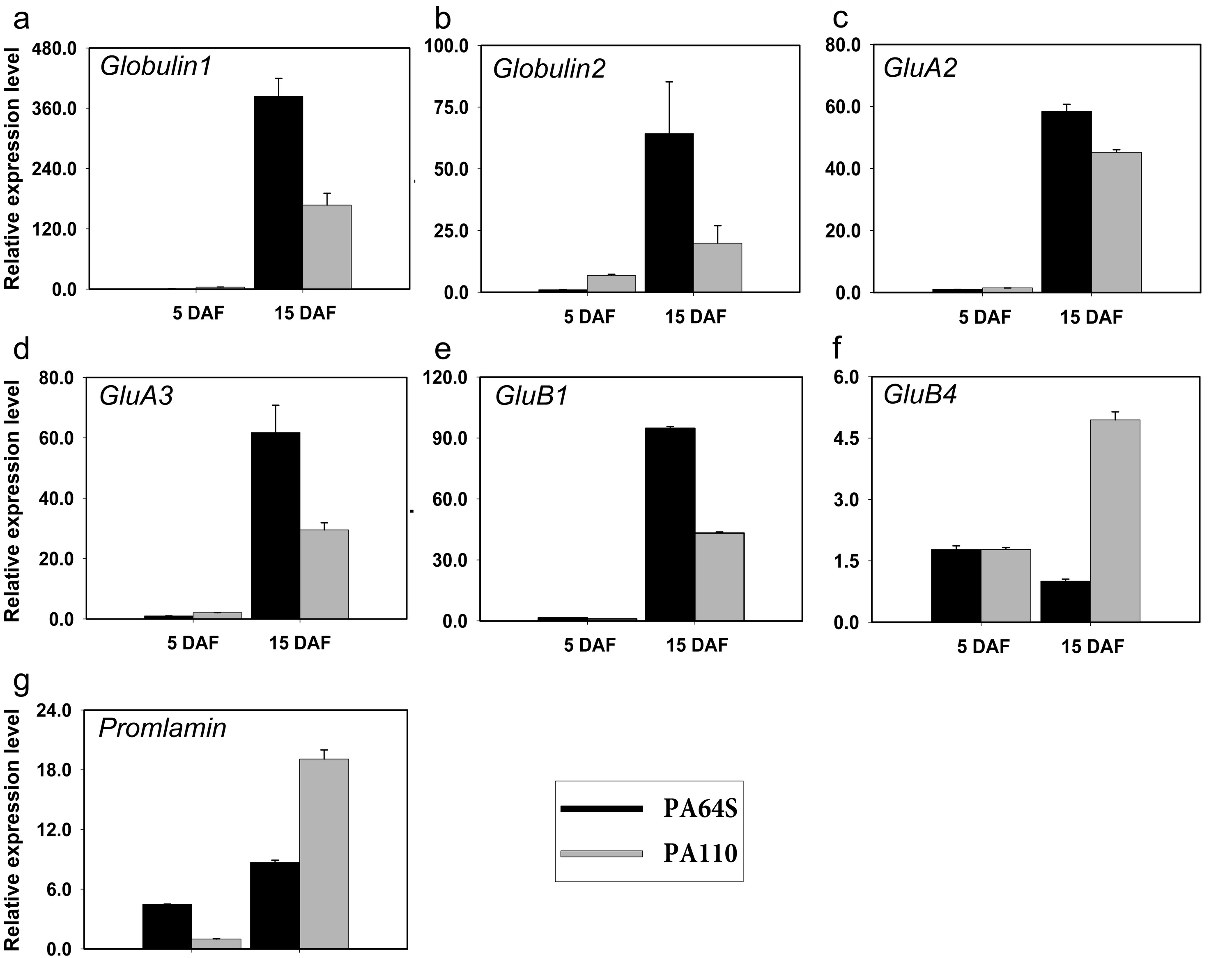

Supplement: Additional file 9: Figure S5. — qRT-PCR analysis of rice genes involved in grain protein biosynthesis. Error bar indicates s.e.m. calculated from 3 technical replicates. (JPG 610 kb) [file 12870_2016_837_MOESM9_ESM.jpg]
